# Supplementary material for: Epidemiological, clinical, and genomic landscape of coccidioidomycosis in northeastern Brazil
Source: Nat Commun. 2024 Apr 12;15:3190. doi: 10.1038/s41467-024-47388-0 (PMC11014852; doi:10.1038/s41467-024-47388-0)
Supplement: Supplementary file 1 — Supplementary Information [file 41467_2024_47388_MOESM1_ESM.pdf]

Inclusion criteria – Patients showing clinical respiratory signs were included, irrespective of extra-pulmonary or hypersensitivity manifestations. Ten patients were subject of previous publications[1-6]. All patients with a clinical diagnosis of coccidioidomycosis-disease (with respiratory signs and/or symptoms, with or without extra-pulmonary dissemination lesions and/or hypersensitivity manifestations) originating from the states of Piauí or Maranhão, and with mycologically confirmed disease by any of the following methods were included in the case series: 1) Identification of mature spherules by direct microscopic examination of any patient's sample in preparations with 10% KOH; 2) Culture of the clinical samples in Sabouraud agar (Becton, Dickinson - BBL) with chloramphenicol supplemented or not with cycloheximide (Sigma-Aldrich) up to 6 weeks at 30°C; positive cultures underwent microscopic identification for hyaline mycelial producing arthroconidial cells; 3) Histopathological examination of biopsies of lung or integumentary tissue with hematoxylin eosin, PAS and Grocott's silver impregnation. The finding of mature spherules was considered a definitive diagnosis. No patients had a prior history suggestive of immunedeficiency such as recurrent infections, immune-mediated diseases, and any illness requiring immunosuppressive regimens. Patients suspected for tuberculosis were tested either by sputum test (bacilloscopy), mycobacterial culture or molecular test (GeneXpert®, Cepheid). The patients' age, sex, ethnicity, occupation, education, and place of origin were determined based on self-report. Risk activities for acquiring *C. posadasii* associated with exposure to soil dust were investigated, such as excavating armadillo burrows during hunting these animals, digging wells, quarrying, excavation at archaeological sites, planting and harvesting agricultural products, recreational activities, road construction, and others. Patients diagnosed and their families were requested to refer present partners and participants in the risk activity for investigation if they presented similar symptoms compatible with coccidioidomycosis. In cases of exposure to armadillo habitat dust, the participation and illness of dogs were also investigated. The affected organs and the presented signs and symptoms were identified in 100 that have more robust clinical data: fever, cough, rash, anorexia, chest pain, dyspnea, weight loss, hemoptysis, myalgia, headache, arthralgia, erythema nodosum, erythema multiforme, skin rash, and others. The date of symptom onset was recorded, and the disease's course duration was calculated from the onset of symptoms to the confirmatory diagnosis of mycosis. In cases with well-defined risk exposure, the incubation period of the disease was estimated. The presence of co-morbidities, such as HIV/AIDS infection, Diabetes Mellitus, tuberculosis, leukemia, lymphomas, other neoplasms, collagen diseases, smoking, alcoholism, and others, was investigated.

Coccidioidomycosis in dogs – Dogs exposed to dust from armadillo habitats during hunting were investigated and managed according to the veterinary practices as described elsewhere[7]. Symptoms of coccidioidomycosis in dogs such as lameness, weakness, fever, fatigue, diminished appetite, weight loss, coughing, and joint discomfort, as well as pain in the back and neck were observed. Disseminated disease was also observed throughout the body since the dogs may exhibit seizures or suffer from vision loss due to the involvement of the central nervous system. The definitive diagnosis relied on isolation of the pathogen in microbiological cultures, by cytological analysis of tissue samples or direct microscopic examination of dog sputum samples in preparations with 10% KOH.

Coccidioides niche modeling and relationship between climate and coccidioidomycosis cases in Brazil - Prevalence density was calculated by collecting the number of cases by municipality and dividing by the area of the municipality (in km2) multiplied by 1,000. We initially utilized 19 bioclimatic variables obtained from WorldClim database at a resolution of 2.5 km (www.worldclim.org/current)[8] as predictors, testing for autocorrelation and selecting variables with r<0.80[9]. Variables were assessed for collinearity with a pairwise Spearman's correlation followed by leave-one-out Jackknife test among all correlated variables in order to rule out the correlated variable that least decreased the model's performance. Model performance was assessed using the area under the curve (AUC) of the receiver operating characteristic (ROC). The final set of variables included Temperature Seasonality, Precipitation Seasonality, Precipitation during the coldest quarter, and Precipitation during the warmest quarter. The average final map had a logistic output, with suitability values ranging from 0 (unsuitable habitat) to 1 (suitable habitat). We employed the machine learning Maxent model ("dismo" R package [10] with 63 presence points and 10,063 total points to determine the Maxent distribution, resulting in an AUC of 0.87 using 1,000 maximum iterations. The model was able to discriminate between positive and negative sites for the presence of *Coccidioides* relatively well (AUC=0.87) and had a greater sensitivity than specificity (AUC=0.926). The average final map had a logistic output, with suitability values ranging from 0 (unsuitable habitat) to 1 (suitable habitat). We examined cases from 1978-2020 in the Northeastern Brazilian states of Maranhão and Piauí to investigate the relationship between climate and coccidioidomycosis cases in Brazil [11], we used negative-binomial regression models (MASS R package[12]), as we are under the assumption that the VF cases are following a negative binomial distribution, with predictor variables of total yearly precipitation, average yearly temperature, and total precipitation of the previous year, and state using the log-link function. The best model was selected based on the lowest AIC. The final model components is as follows:

$$\ln(Y_{it}) = \beta_0 + \beta_1(avg.\ yearly\ temp_{it}) + \beta_2(total\ precip\ in\ year\ t_{it}) + \beta_3(total\ precip\ in\ year\ t-1_{it-1}) + \beta_4(state_i) + \varepsilon.$$

Where Y_{it} is the number of coccidioidomycosis cases in state i and year t

We checked for autocorrelation among the predictor variables in the model using a correlation matrix with the GGally R package[13]. All variables that were kept in the model had Person's correlation coefficients <0.6.

Physicochemical characteristics of the soil samples - We collected nine soil samples from sites where patients reported high levels of dust exposure before experiencing clinical symptoms (e.g., armadillo burrows, quarries, or charcoal factories). The samples were aseptically collected in containers with screw caps, ensuring an airtight seal. We collected approximately 1000 g of soil from each site, selecting samples from at least six different points within a depth range of 10 to 30 cm below the soil surface. Subsequently, we measured the soil samples' physicochemical characteristics, including texture, salinity, pH, and total organic content, using methods described elsewhere[14]. The

analyses were conduced at the Laboratório de Analise de Solos (LASO), Federal University of Piauí. This soils were categorized according to the amount or organic matter content based on the USDA (United States Department of Agriculture) criteria[15]as follows: Low Organic Matter Content: Soil with organic carbon content below 1-2% is considered to have low organic matter content. Medium Organic Matter Content: Soil with organic carbon content between 2-3.5% might be categorized as having medium organic matter content. High Organic Matter Content: Soil with organic carbon content above 3.5-6% or higher can be considered to have high organic matter content.

DNA sequencing and evolutionary analysis - Fungal growth was achieved by plating clinical specimens (see above) into the fungibiotic media Mycosel (Becton, Dickinson - BBL). Whole genome typing was performed in 13 isolates since the majority of the isolates were discarded after the confirmation of *Coccidioides* sp. diagnosis due to the lack of BLS-3 laboratory in both Institute of Tropical Diseases Nathan Portela and at the Pulmonology Clinic of Hospital Getúlio Vargas in the Piaui state. Thus, all live strains were all transferred to the National Reference Mycology Laboratory at the Oswaldo Cruz Foundation (Fiocruz), in Rio de Janeiro for molecular typing. DNA was extracted from 500mg of cells[16], assessed for integrity via agarose gel electrophoresis, 1μg of input DNA subjected to libraries preparation using the NEBNext® Ultra™ II DNA Library Prep Kit (New England Biolabs) and quantified via qPCR, Bionalyzer (Agilent) and Qubit (Invitrogen). DNA libraries were sequenced using the NovaSeq 6000 instrument (Illumina), kit v1.5 - 300 cycles - 2X150bp, in a high-throughput mode. Initial quality control for the sequenced reads were performed using the FastQC v0.11.9 pipeline[17]. We mapped the Illumina fastq reads into the reference *C. posadasii* strain Silveira[18] (https://www.ncbi.nlm.nih.gov/datasets/genome/GCA_018416015.2/) using bwa-mem v0.7.7 [19]. We have also mapped 81 additional *Coccidioides* sp. genomes to the reference *C. posadasii* Silveira strain for evolutionary comparisons; read samples are available under SRA experiments SRP148748 (https://www.ncbi.nlm.nih.gov/Traces/study/?acc=SRP148748&o=acc_s%3Aa), SRP135537 (https://www.ncbi.nlm.nih.gov/Traces/study/?acc=SRP135537&o=acc_s%3Aa) and SRP074212 (https://www.ncbi.nlm.nih.gov/Traces/study/?acc=SRP074212&o=acc_s%3Aa). Next, we identified mismatch intervals and indels with help of GATK v3.3 tools RealignerTargetCreator and IndelRealigner[20]. We utilized the GATK UnifiedGenotyper, a part of GATK toolkit, using the parameter het = 0.01 to account for a haploid organism to retrieve SNPs. We used the following filters to obtain high-confident SNP calls: QD = 2 || FS_filter = 60 || MQ_filter = 30 || MQ_Rank_Sum_filter = -12.5 || Read_Pos_Rank_Sum_filter = -8 (see [21]). We purged SNPs with less than 10X coverage, with less than 90% variant allele calls, or that were identified by Nucmer v3.23[22] as located in duplicated regions in the reference genome. We measured nucleotide diversity of the strains from Brazil using the Maximum likelihood composition method available in the MEGA X software [23]. The retrieved 502,553 SNPs across 94 taxa were submitted to phylogenomic analysis using IQTREE2[24] software using the concatenation approach. We used ModelFinder [25] to calculate the best nucleotide substitution model under the Bayesian Information Criterion. The best tree topology was calculated under the

Maximum Likelihood criteria and branch support was assessed using both SH-like alternate Likelihood Ratio Test (aLRT[26]) and ultrafast bootstraps with 1,000 replicates[27]. The tree topology was visualized using the FigTree v1.4.4 (http://tree.bio.ed.ac.uk/software/figtree/). Principal Coordinate Analysis (PCA) was applied via the R package adegenet[28] to evaluate population structure. The resulting Eigenvalues were used to calculate the two main prominent variations (PC1 and PC2) and to verify the population splits within *C. posadasii*.

Data availability:
Bioproject: PRJNA1000610
Biosample: SAMN36772057-SAMN36772069
SRA deposit: SRR25495556-SRR25495568

1 Deus Filho, A., Rocha Filho, Z. & Wanke, B. Microepidemia de coccidioidomicose em caçadores de tatu na cidade de Floriano no estado do Piauí. *J. Pneumol.* **26**, 2 (2000).

2 Moraes, M. A., Martins, R. L., Leal, II, Rocha, I. S. & Medeiros Junior, P. [Coccidioidomycosis: a new brazilian case]. *Rev Soc Bras Med Trop* **31**, 559-562, doi:10.1590/s0037-86821998000600009 (1998).

3 Martinez, R. Coccidioidomicose no Brasil: relato de novo caso. *Rev Soc Bras Med Trop* **35**, 1 (2002).

4 Veras, K. N., Figueiredo, B. C. S., Martins, L. M. S., Vasconcelos, J. T. P. & Wanke, B. Coccidioidomicose: causa rara de síndrome do desconforto respiratório agudo. *J Pneumol* **29**, 4 (2003).

5 Vianna, H., Passos, H. V. & Sant'ana, A. V. [Coccidioidomycosis. Report of the 1st case in a native of Brazil]. *Rev Inst Med Trop Sao Paulo* **21**, 51-55 (1979).

6 Wanke, B. *et al.* Investigation of an outbreak of endemic coccidioidomycosis in Brazil's northeastern state of Piaui with a review of the occurrence and distribution of Coccidioides immitis in three other Brazilian states. *Mycopathologia* **148**, 57-67 (1999).

7 Graupmann-Kuzma, A. *et al.* Coccidioidomycosis in dogs and cats: a review. *Journal of the American Animal Hospital Association* **44**, 226-235 (2008).

8 Hijmans, R. J., Cameron, S. E., Parra, J. L., Jones, P. G. & Jarvis, A. Very high resolution interpolated climate surfaces for global land areas. *International Journal of Climatology* **25**, 1965-1978, doi:https://doi.org/10.1002/joc.1276 (2005).

9 Hernandez, P. A., Graham, C. H., Master, L. L. & Albert, D. L. The effect of sample size and species characteristics on performance of different species distribution modeling methods. *Ecography* **29**, 773-785, doi:https://doi.org/10.1111/j.0906-7590.2006.04700.x (2006).

10 Elith*, J. *et al.* Novel methods improve prediction of species' distributions from occurrence data. *Ecography* **29**, 129-151, doi:https://doi.org/10.1111/j.2006.0906-7590.04596.x (2006).

11 Camarillo-Naranjo, J. M., Álvarez-Francoso, J. I., Limones-Rodríguez, N., Pita-López, M. F. & Aguilar-Alba, M. The global climate monitor system: from climate data-handling to knowledge dissemination. *International Journal of Digital Earth* **12**, 394-414, doi:10.1080/17538947.2018.1429502 (2019).

12 Venables, B. & Ripley, B. (2002).
13 GGally: Extension to 'ggplot2' (2022).
14 Teixeira, P. C., Donagemma, G. K., Fontana, A. & Teixeira, W. *Manual de métodos de análise de solo.* 3rd edn, (Embrapa Solos, 2017).
15 Ditzler, C., Scheffe, K. & Monger, H. C. *Soil survey manual, USDA Handbook 18*. (Government Printing Office, 2017).
16 Muniz Mde, M., Morais, E. S. T. P., Meyer, W., Nosanchuk, J. D. & Zancope-Oliveira, R. M. Comparison of different DNA-based methods for molecular typing of Histoplasma capsulatum. *Appl Environ Microbiol* **76**, 4438-4447, doi:10.1128/AEM.02004-09 (2010).
17 FastQC: a quality control tool for high throughput sequence data (http://www.bioinformatics.babraham.ac.uk/projects/fastqc, 2010).
18 de Melo Teixeira, M. *et al.* A chromosomal-level reference genome of the widely utilized Coccidioides posadasii laboratory strain “Silveira”. *G3 Genes|Genomes|Genetics* **12**, jkac031, doi:10.1093/g3journal/jkac031 (2022).
19 Li, H. & Durbin, R. Fast and accurate short read alignment with Burrows-Wheeler transform. *Bioinformatics* **25**, 1754-1760, doi:10.1093/bioinformatics/btp324 (2009).
20 McKenna, A. *et al.* The Genome Analysis Toolkit: a MapReduce framework for analyzing next-generation DNA sequencing data. *Genome Res* **20**, 1297-1303, doi:10.1101/gr.107524.110 (2010).
21 Teixeira, M. M. *et al.* Population Structure and Genetic Diversity among Isolates of Coccidioides posadasii in Venezuela and Surrounding Regions. *mBio* **10**, doi:10.1128/mBio.01976-19 (2019).
22 Kurtz, S. *et al.* Versatile and open software for comparing large genomes. *Genome Biol* **5**, R12, doi:10.1186/gb-2004-5-2-r12 (2004).
23 Stecher, G., Tamura, K. & Kumar, S. Molecular Evolutionary Genetics Analysis (MEGA) for macOS. *Mol Biol Evol* **37**, 1237-1239, doi:10.1093/molbev/msz312 (2020).
24 Minh, B. Q. *et al.* IQ-TREE 2: New Models and Efficient Methods for Phylogenetic Inference in the Genomic Era. *Mol Biol Evol* **37**, 1530-1534, doi:10.1093/molbev/msaa015 (2020).
25 Kalyaanamoorthy, S., Minh, B. Q., Wong, T. K. F., von Haeseler, A. & Jermiin, L. S. ModelFinder: fast model selection for accurate phylogenetic estimates. *Nat Methods* **14**, 587-589, doi:10.1038/nmeth.4285 (2017).
26 Anisimova, M. & Gascuel, O. Approximate likelihood-ratio test for branches: A fast, accurate, and powerful alternative. *Syst Biol* **55**, 539-552, doi:10.1080/10635150600755453 (2006).
27 Minh, B. Q., Nguyen, M. A. & von Haeseler, A. Ultrafast approximation for phylogenetic bootstrap. *Mol Biol Evol* **30**, 1188-1195, doi:10.1093/molbev/mst024 (2013).
28 Jombart, T. & Ahmed, I. adegenet 1.3-1: new tools for the analysis of genome-wide SNP data. *Bioinformatics* **27**, 3070-3071, doi:10.1093/bioinformatics/btr521 (2011).

Supplementary Figure 1

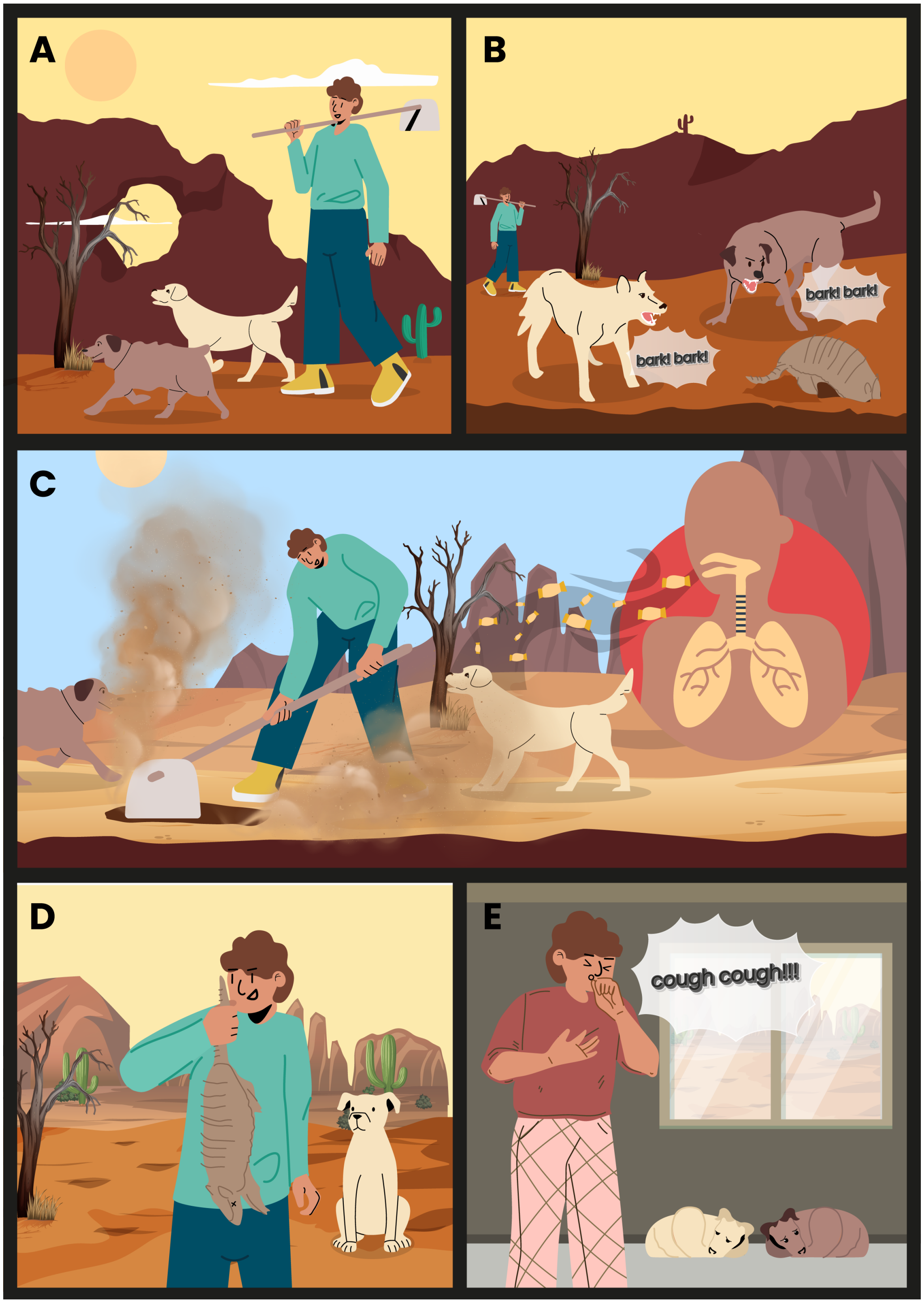

Supplementary Figure 1 – Coccidioidomycosis acquired during armadillo hunting practices in Northeastern Brazil. A) Armadillo hunting is a centenary practice in Brazil that use speed chasing and ambush by hunters and dogs, and is the most common

practice for acquiring coccidioidomycosis in the Brazilian semiarid region. B) Hunting dogs smell the armadillos hundreds of meters away and chase the armadillos, which digs into its burrow to escape. The hunting dogs start to bark, alerting hunters. C) Once located, the hunter excavates the armadillo from its burrow with help of a hoe and shovel. Soil particles become aerosolized, and a large volume of dust is formed potentially containing infectious arthroconidia that can be inhaled by humans and dogs. D) After a lengthy excavation, the armadillo is finally extracted from its burrow and take to the hunter's home to be consumed. E) Around 30 days after exposure, both humans and dogs develop the initial symptoms of acute coccidioidomycosis, such as cough, fever and chest pain.
