# Supplementary material for: Epidemiological, clinical, and genomic landscape of coccidioidomycosis in northeastern Brazil
Source: Nat Commun. 2024 Apr 12;15:3190. doi: 10.1038/s41467-024-47388-0 (PMC11014852; doi:10.1038/s41467-024-47388-0)
Supplement: Supplementary file 3 — Reporting Summary [file 41467_2024_47388_MOESM3_ESM.pdf]

nature portfolio

Corresponding author(s): MMT - NCOMMS-23-39654D

Last updated by author(s): Mar 11, 2024

Reporting Summary

Nature Portfolio wishes to improve the reproducibility of the work that we publish. This form provides structure for consistency and transparency in reporting. For further information on Nature Portfolio policies, see our Editorial Policies and the Editorial Policy Checklist.

Statistics

For all statistical analyses, confirm that the following items are present in the figure legend, table legend, main text, or Methods section.

n/a	Confirmed	
☐	☒	The exact sample size (*n*) for each experimental group/condition, given as a discrete number and unit of measurement
☐	☒	A statement on whether measurements were taken from distinct samples or whether the same sample was measured repeatedly
☐	☒	The statistical test(s) used AND whether they are one- or two-sided<br>*Only common tests should be described solely by name; describe more complex techniques in the Methods section.*
☐	☒	A description of all covariates tested
☐	☒	A description of any assumptions or corrections, such as tests of normality and adjustment for multiple comparisons
☐	☒	A full description of the statistical parameters including central tendency (e.g. means) or other basic estimates (e.g. regression coefficient) AND variation (e.g. standard deviation) or associated estimates of uncertainty (e.g. confidence intervals)
☐	☒	For null hypothesis testing, the test statistic (e.g. *F*, *t*, *r*) with confidence intervals, effect sizes, degrees of freedom and *P* value noted<br>*Give P values as exact values whenever suitable.*
☒	☐	For Bayesian analysis, information on the choice of priors and Markov chain Monte Carlo settings
☒	☐	For hierarchical and complex designs, identification of the appropriate level for tests and full reporting of outcomes
☒	☐	Estimates of effect sizes (e.g. Cohen's *d*, Pearson's *r*), indicating how they were calculated

Our web collection on statistics for biologists contains articles on many of the points above.

Software and code

Policy information about availability of computer code

Data collection	The reference C. posadasii strain Silveira was downloaded for the following URL: https://www.ncbi.nlm.nih.gov/datasets/genome/GCA_018416015.2/. Additional 81 Coccidioides sp. genomes were used for evolutionary comparisons; read samples are available under SRA experiments SRP148748 (https://www.ncbi.nlm.nih.gov/Traces/study/?acc=SRP148748&o=acc_s%3Aa), SRP135537 (https://www.ncbi.nlm.nih.gov/Traces/study/?acc=SRP135537&o=acc_s%3Aa) and SRP074212 (https://www.ncbi.nlm.nih.gov/Traces/study/?acc=SRP074212&o=acc_s%3Aa).
Data analysis	We used open source softwares to perform statistical, PCA (tool Adegenet 1.3.1) and species niche modeling analysis (tools 'sf', 'spatstat', 'stars' and 'MASS') are implemented in the R package version 4.2. For genomic analyses: Read quality control - FastQC v0.11.9; Read alignment - bwa-mem v 0.7.7; SNP calls - GATK v3.3; genome alignments - Nucmer v3.23; nucleotide diversity - MEGA X software; Phylogenomic trees - IQTREEv2; Tree visualization - FigTree v1.4.4;

For manuscripts utilizing custom algorithms or software that are central to the research but not yet described in published literature, software must be made available to editors and reviewers. We strongly encourage code deposition in a community repository (e.g. GitHub). See the Nature Portfolio guidelines for submitting code & software for further information.

Data

Policy information about availability of data

All manuscripts must include a data availability statement. This statement should provide the following information, where applicable:

- Accession codes, unique identifiers, or web links for publicly available datasets
- A description of any restrictions on data availability
- For clinical datasets or third party data, please ensure that the statement adheres to our policy

Due to privacy and ethical considerations, the raw data cannot be openly shared. For inquiries regarding data access or specific requests for further information, please contact the corresponding author. We are committed to facilitating access to the extent possible while respecting the confidentiality and privacy of the individuals involved in this research. The 19 WorldClim Bioclimatic variables are available at https://www.worldclim.com/version2. The reference C. posadasii strain Silveira was downloaded for the following URL: https://www.ncbi.nlm.nih.gov/datasets/genome/GCA_018416015.2/. Additional 81 Coccidioides sp. genomes were used for evolutionary comparisons; read samples are available under SRA experiments SRP148748 (https://www.ncbi.nlm.nih.gov/Traces/study/?acc=SRP148748&o=acc_s%3Aa), SRP135537 (https://www.ncbi.nlm.nih.gov/Traces/study/?acc=SRP135537&o=acc_s%3Aa) and SRP074212 (https://www.ncbi.nlm.nih.gov/Traces/study/?acc=SRP074212&o=acc_s%3Aa).
Rainfall data were collected from the public repository of the Instituto Nacional de Meteorologia (https://portal.inmet.gov.br/)

Research involving human participants, their data, or biological material

Policy information about studies with human participants or human data. See also policy information about sex, gender (identity/presentation), and sexual orientation and race, ethnicity and racism.

Reporting on sex and gender	The authors use the terms sex for biological attribute by the participants based on a questionnaire
Reporting on race, ethnicity, or other socially relevant groupings	Ethnic identity was determined by the participants based on a questionnaire
Population characteristics	Age, sex and other population characteristics were determined by the participants based on a questionnaire. This information is presented in Table 1
Recruitment	Patients were referred to the hospital presenting symptoms with coccidioidomycosis
Ethics oversight	Ethics Committee of the Federal University of Piaui (No.36/08 - CAAE 0036.0.045.000-8) - This information was added to the manuscript

Note that full information on the approval of the study protocol must also be provided in the manuscript.

Field-specific reporting

Please select the one below that is the best fit for your research. If you are not sure, read the appropriate sections before making your selection.

☒ Life sciences ☐ Behavioural & social sciences ☐ Ecological, evolutionary & environmental sciences

For a reference copy of the document with all sections, see nature.com/documents/nr-reporting-summary-flat.pdf

Life sciences study design

All studies must disclose on these points even when the disclosure is negative.

Sample size	292 individuals - Our aim was to gather as many retrospective cases a we could and describe them so there was no sample size calculation. The 292 cases give us enough statistical power to estimate the proportions of the main features described in the present paper (armadillo hunting, pulmonary clinical presentation). Indeed, less than a hundred would yield sufficient power to measure the prevalences with a 5% precision given our sample. Hence, our main messages are supported by sufficient power and our efforts for exhaustive collection of clinical cases.
Data exclusions	192 that haven't detailed and complete clinical data
Replication	No replicates was used since this was a epidemiological retrospective and descriptive study. Retrospective studies analyze historical data, and while researchers can perform sensitivity analyses or validate findings using different datasets, true replication is challenging because the events have already occurred.
Randomization	This is not relevant since this was a retrospective study. In retrospective studies, researchers are analyzing data from past events, and there is no opportunity for random assignment. The groups are often formed based on existing conditions or exposures, making randomization impractical.
Blinding	This is not relevant since this was a epidemiological retrospective study. In retrospective studies, data are collected after events have taken

Blinding	place, and there is typically no direct interaction with participants. Blinding is less relevant because the exposure or intervention status is often known, and biases related to participant or researcher awareness are less likely.

Reporting for specific materials, systems and methods

We require information from authors about some types of materials, experimental systems and methods used in many studies. Here, indicate whether each material, system or method listed is relevant to your study. If you are not sure if a list item applies to your research, read the appropriate section before selecting a response.

Materials & experimental systems

n/a	Involved in the study	
☒	☐	Antibodies
☒	☐	Eukaryotic cell lines
☒	☐	Palaeontology and archaeology
☒	☐	Animals and other organisms
☐	☒	Clinical data
☒	☐	Dual use research of concern
☒	☐	Plants

Methods

n/a	Involved in the study	
☒	☐	ChIP-seq
☒	☐	Flow cytometry
☒	☐	MRI-based neuroimaging

Clinical data

Policy information about clinical studies

All manuscripts should comply with the ICMJE guidelines for publication of clinical research and a completed CONSORT checklist must be included with all submissions.

Clinical trial registration	NA since this was a epidemiological retrospective and descriptive study
Study protocol	NA
Data collection	We conducted a retrospective study of microbiologically proven coccidioidomycosis cases between 1978 to 2021 at the Institute of Tropical Diseases Nathan Portela and at the Pulmonology Clinic of Hospital Getulio Vargas. In this study, the patients were referred from Maranh5o and Piaui states of Brazil. Patients showing clinical respiratory signs were evaluated and inclusion criteria were based on the isolation or observation of fungal cells compatible with Coccidioides
Outcomes	Lung damage was assessed using chest X-ray and/or computed tomography (CT). Residual lesions such as nodules or cavitation and sequelae such as calcifications, fibrosis or bronchiectasis were investigated. Cure was defined according to the https://doi.org/10.1093/cid/ciw360

Plants

Seed stocks	NA
Novel plant genotypes	NA
Authentication	NA
